# Cost-Utility Analysis of Oxybutynin vs. OnabotulinumtoxinA (Botox) in the Treatment of Overactive Bladder Syndrome

**DOI:** 10.3390/ijerph18168743

**Published:** 2021-08-19

**Authors:** Habiba Shabir, Sana Hashemi, Moussa Al-Rufayie, Tayo Adelowo, Umar Riaz, Umayair Ullah, Benyamin Alam, Mehreen Anwar, Laure de Preux

**Affiliations:** 1 Department of Management and Entrepreneurship, Business School, Imperial College London, London SW7 2BX, UK; k1765319@kcl.ac.uk (S.H.); moussa.al-rufayie17@imperial.ac.uk (M.A.-R.); tayoadelowo@googlemail.com (T.A.); umar.riaz20@imperial.ac.uk (U.R.); umayair.ullah20@imperial.ac.uk (U.U.); mehreen_anwar@hotmail.co.uk (M.A.); 2 Faculty of Biology, Medicine and Health, University of Manchester, Manchester M13 9PL, UK; benyamin.alam@student.manchester.ac.uk; 3 Department of Economics and Public Policy, Business School, Imperial College London, London SW7 2BX, UK; l.depreux@imperial.ac.uk

**Keywords:** economic evaluation, cost-utility, oxybutynin, onobotulinumtoxinA, overactive bladder syndrome

## Abstract

*Background:* The UK National Health Service (NHS) propose the use of oxybutynin prior to onabotulinumtoxinA (Botox) in the management of overactive bladder syndrome (OAB). Oxybutynin is costly and associated with poor adherence, which may not occur with Botox. We conducted a cost-utility analysis (CUA) to compare the medications. *Methods:* we compared the two treatments in quality-adjusted life years (QALYS), through the NHS’s perspective. Costs were obtained from UK-based sources and were discounted. Total costs were determined by adding the treatment cost and management cost for complications on each branch. A 12-month time frame was used to model the data into a decision tree. *Results:* Our results found that using Botox first-line had greater cost utility than oxybutynin. The health net benefit calculation showed an increase in 0.22 QALYs when Botox was used first-line. Botox also had greater cost-effectiveness, with the exception of pediatric patients with an ICER of £42,272.14, which is above the NICE threshold of £30,000. *Conclusion:* Botox was found to be more cost-effective than antimuscarinics in the management of OAB in adults, however less cost-effective in younger patients. This predicates the need for further research to ascertain the age at which Botox becomes cost-effective in the management of OAB.

## 1. Introduction

### 1.1. Background

Overactive bladder syndrome, or OAB, has been defined as “urgency, with or without urge incontinence, usually with frequency and nocturia” [1]. Patients often experience a sudden urge to urinate, especially at night, which can severely impact an individual’s quality of life, mental health, and personal relationships [2]. Moreover, OAB has been associated with an increased risk of falls in the elderly, as they hurry to the restroom more often [3].

It has been estimated that roughly 12% of the total adult population (over 18) suffers from OAB, affecting men and women equally [4]. In children, OAB is the most common cause of voiding issues, which negatively affects both the child and the rest of the family [5]. Paediatric OAB can also continue in adulthood, making it harder to treat [5]. The aetiology of OAB is most likely multifactorial; neurological dysfunction, bladder outlet obstruction, and genetics have all been implicated in the hunt for the cause [6]. Additionally, a significant number of cases are idiopathic in origin [6].

Initially, the management of OAB consisted of lifestyle changes, such as reducing caffeine and alcohol intake alongside exercises, for instance pelvic floor exercises and bladder training [7]. Currently, the first-line treatment, after lifestyle changes, is the administration of anticholinergic/antimuscarinic agents, such as oxybutynin [7]. According to NICE guidelines, other anticholinergics will be offered if the initial drug does not work, followed by mirabegron if anticholinergics are unsuitable for the patient [7].

In cases where the above therapies are not successful, NICE recommend injecting onabotulinumtoxinA (Botox) into the bladder as one of the second-line treatments [7,8]. The Botox is injected into the detrusor muscle of the bladder in multiple locations via a cystoscope under local or general anaesthesia [9]. Botulinum toxin blocks the release of acetylcholine at the neuromuscular junction, preventing muscle contraction [10]. Unlike other medications, onabotulinumtoxinA is not covered by the PbR tariff [11]. Patients who have not responded adequately to other treatments must be referred to the PCT by their MDT for exceptional funding [11]. However, while antimuscarinics are cheaper than Botox administration, certain side-effects, such as dry mouth, constipation, and dizziness, have contributed to patients discontinuing the treatment after a while [12].

### 1.2. Motivation and Rationale

OAB is a condition that affects the adult and pediatric population significantly, both mentally and physically [13]. The total direct economic impact on the NHS is almost £900 million, and the estimated costs per patient per annum were higher in the UK compared to other countries in the western hemisphere, such as Germany and Sweden [13]. Indirect costs due to absenteeism because of OAB is estimated to be just over £9.5 billion [13].

Low adherence rates in patients prescribed with oxybutynin have been noted; in one study, 67% of participants discontinued their medication after 2 years, with 63% of them doing so in the first 2 months due to side effects [14]. Long-term use of oxybutynin has also been associated with an increased risk of dementia [15], further highlighting the need for alternative therapies with fewer side-effects.

While the next line of therapy after antimuscarinics is mirabegron, a recent US cost-effectiveness study has demonstrated onabotulinumtoxinA’s superiority in relation to Mirabegron, as well as non-oxybutynin antimuscarinics [16]. Therefore, we decided to take this a step further and perform an economic analysis to gain a better understanding of the cost-utility of Botox and oxybutynin, to make the OAB treatment plan more effective. We believe that Botox should be offered as a first-line treatment.

### 1.3. Study Objectives

The aim of this evaluation is to compare the cost-utility of using oxybutynin against onabotulinumtoxinA for the treatment of overactive bladder syndrome. Recommendations on how best to allocate the limited resources will be made using the findings of this analysis.

### 1.4. Literature Review

**Figure 1:**
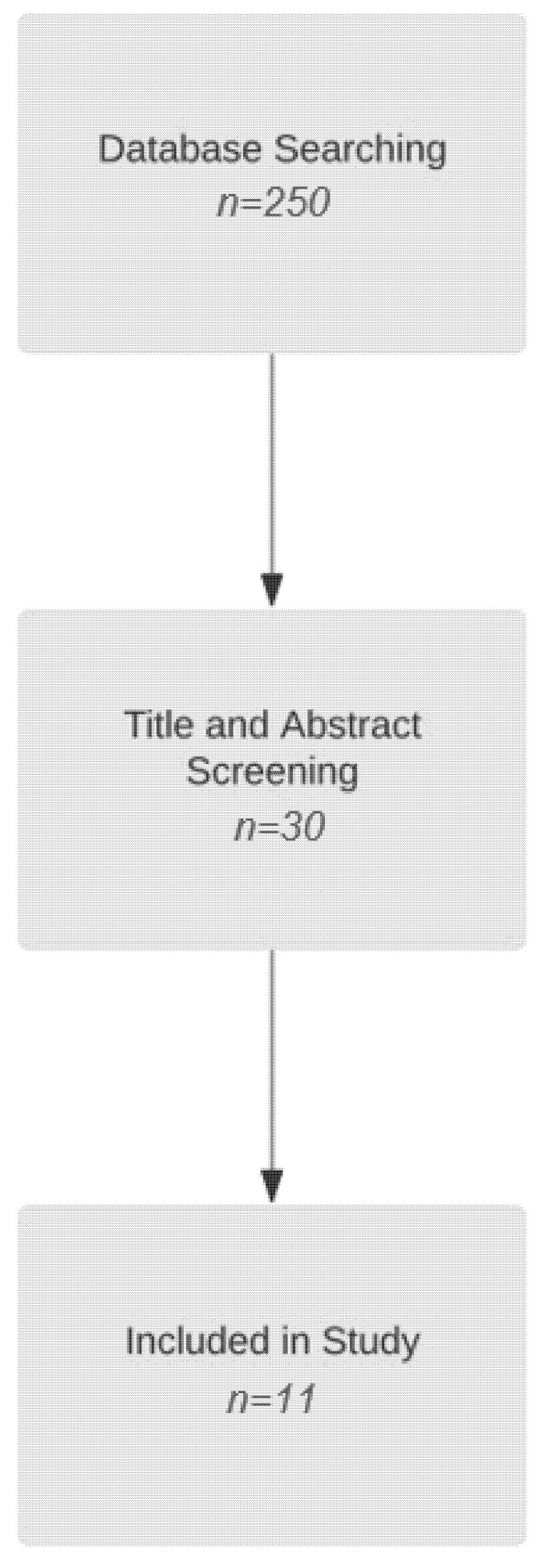
Studies used in NLR.

A narrative literature review using Google Scholar, PubMed, NICE, and Cochrane databases was performed to gain a better understanding of previous analyses and to obtain the necessary data for the CUA. Key search terms included: ‘onabotulinumtoxinA’, ‘botox’, ‘oxybutynin’, ‘overactive bladder’, ‘anticholinergics’, ‘antimuscarinics’, ‘cost-effectiveness’, ‘cost-analysis’, and ‘cost-benefit’. Our search did not yield any results for a cost-utility analysis comparing the two drugs. Strict inclusion and exclusion criteria (Appendix F) helped us limit selection bias, which became a key part in the screening stage of our NLR. The number of papers included after each stage of screening can be seen in Figure 1.

Other cost data that could not be obtained through the literature review were collected from NHS, BNF, and PSSRU databases.

As a first-line treatment, the effectiveness of oxybutynin has been well established [17]. A RCT has demonstrated the efficacy of Botox in treating detrusor overactivity compared with placebo [18]. Another RCT also highlighted the effectiveness of Botox in treating idiopathic OAB [19]. Network meta-analysis discovered that Botox is more effective at symptom relief compared to other agents, including oxybutynin [20].

A cost-effectiveness analysis in the US found that Botox was more effective in treating detrusor overactivity compared to best supportive care (BSC), a treatment package that includes anticholinergics, incontinence pads, or catheterization [21]. Another CEA comparing Botox and BSC arrived at the same conclusion [22]. A US-based CEA advocated for the use of Botox as a first-line treatment, after comparing it to tolterodine and Solifenaicn [23]. Botox was also found to be cost-effective against sacral neuromodulation, an implanted device that corrects dysfunctional signaling along the nerves [24].

The effectiveness of Botox has been demonstrated by many studies, setting up the stage to conduct a cost-utility analysis. Twelve key articles were shortlisted to obtain data. Cost data were collected from three main databases: NHS cost data collections [25], the BNF [26,27,28,29], the Personal Social Services Research Unit [30], and two CEA’s 22, 23 (Table A1, Table A2, Table A3, Table A4 and Appendix B). QALYs were calculated using data from the *European Journal of Health Economics* article [22] (Appendix D and Appendix E) and probabilities were obtained from five studies [22,23,31,32,33] (List of studies in Appendix C).

## 2. Methods

Given the apparent scarcity of existing literature directly comparing the two management options, it is paramount that further evaluation is conducted to elucidate which option is superior and possesses greater feasibility. As no current research has been conducted, our analysis could not be modelled off existing trials. As a result, we utilized a standardized approach to ensure accurate and valid results

The costs of the two treatments were extracted from NHS national tariffs and reference costing alongside the BNF and published articles

### 2.1. Choice of Analysis

A cost-utility analysis (CUA) was chosen for this evaluation, as it was important to determine the impact of the new treatment on the patient’s quality of life. A CUA measures the overall utility of the intervention, which is its value based on the individual’s preferences. This is a useful tool to use for evaluating programmed based on non-monetary terms, giving a more holistic evaluation of the advantages of the new intervention. Quality-adjusted life years are the units used in CUAs to measure the value of health outcomes, including both the quality and quantity of life lived [34]. For example, an increase in 1 QALY means that the patient will live 1 more year in perfect health.

While it is slightly more difficult to appreciate the impact of an intervention on the patient’s quality of life, there are many frameworks that can help us obtain an accurate measure. The incontinence-specific quality of life questionnaire (I-QOL) is a self-assessment questionnaire that measures one’s quality of life specific to urinary problems and their impact. The EQ-5D values will be derived from the EQ-5D questionnaire to help determine the quality of life, as it considers the following dimensions while measuring a patient’s health status: depression/anxiety, pain/discomfort, self-care, usual activities, and mobility [34]. The EQ-5D values will then be translated into QALYs by multiplying the utility value by the value of 1 (the time frame). A cost-utility evaluation is more suited to the NHS, as this method of economic analysis will provide a more holistic view of the benefits of a particular intervention.

### 2.2. Choice of Perspective

This study was conducted through the perspective of the National Health Service (NHS), which is free at the point of delivery. As a taxpayer-funded health care system, the NHS has a fixed and limited budget that must serve the needs of the country whilst maintaining high standards of care. Therefore, precious resources must be allocated efficiently and sustainably, whilst also ensuring that patients are treated fairly and equally.

### 2.3. Costs

Our costs were obtained from the sources mentioned before, and as they are all UK-based costings and were valid to use in our model. The cost layout was determined from a CEA of Botox when compared to BSC from a UK perspective [22] and a CEA comparing antimuscarinics to solifenacin [32]. Costs were discounted according to the NICE rate of 3.5% [35], to avoid underestimating the costs of treatment from our studies. As our raw costing data came from 2 studies conducted in 2016 [22] and 2018 [23], a discount rate of 1.0356 and 1.0353 were applied, respectively, to bring the cost values to 2021 (Appendix B).

#### 2.3.1. Medication Costs

Laxatives are a patient cost, not NHS, as they can be bought over the counter (OTC) [36]. However, we justified including these costs because OTC only occurs in short-term laxative use, and long-term use warrants prescription charges which costs the NHS [37]. As this study spans over a year and constipation is a long-term side effect of oxybutynin, this cost would be relevant from the NHS perspective. In contrast, the cost for dry mouth was set as £0.00 [38], because any sprays/lozenges/gels are OTC products and, therefore, purely a patient cost. Costs for treatments are listed in Table 1.

#### 2.3.2. Physiotherapy

Pelvic muscle training sessions are offered by the NHS before medications [7]. Therefore, they were omitted as our time frame starts from the initiation of drug therapy.

### 2.4. Benefits

The utilities in the study conducted by the *European Journal of Health Economics* were measured using the incontinence-specific quality of life questionnaire (I-QOL) [22]. The I-QOL is a self-assessment questionnaire that measures one’s quality of life specific to urinary problems and their impact. These were converted into EQ-5D values via mapping algorithms based on a study observing UK preferences [39] to form the final utilities we used from the onabotulinumtoxinA study [22].

A disutility of −0.036 per side effect was used to account for the impact of side effects on the patients’ quality of life [32], giving a more accurate representation of the final utilities. The EQ-5D values were equal to the QALYs as the study time span was 1 year, and therefore the multiplication factor was 1. The 2021 values of the QALYs are the same as the data obtained from the 2016 study [22], as there is no discounting of this measurement.

Omitted utilities include those of family members/caregivers who support the patient. UIE is a dehumanizing and embarrassing condition for those who suffer from it and has a negative impact on caregivers [40]. Despite the emotional burden for caregivers, there was lack of concrete utility data that could reflect this.

### 2.5. Modelling

We used the time frame of 12 months as much of our data were collected at this point, including utilities and probabilities. However, this meant the reinjection rate of Botox was excluded after the treatment switch from oxybutynin to Botox. Injections occur every six months, and treatment switches take time to implement [23], therefore, we concluded that within our time frame, reinjections would not arise.

The total costs were determined by adding the costs of treatments and the management of any complications that arose on that branch. The calculations occurred from right to left to find the cost and utility values at each node based on the probabilities. The final outcome at the end of each branch is the number of urinary incontinence episodes per 24 h. This is depicted as 4 levels as seen in the Botox study [22]. The full decision tree is included in the Supplementary Material. The costs and utitily values of complications are demonstrated in Table 2.

## 3. Results

Our results show that using Botox as a first-line treatment had greater cost-utility than oxybutynin. The results indicate that, for every £12,225.68 spent, there is a gain of one QALY. The ICER of £12,225.68 is far below the NICE threshold of 30,000 pounds which is in line with the ABC trial findings from the US perspective, suggesting that Botox is more cost-effective [41]. The monetary net benefit (MNB) and health net benefit (HNB) were performed to convert the ICER into monetary and health terms. The results were both positive, as expected from the ICER. The HNB shows there would be an overall increase in 0.22 QALY in the population if Botox were implemented as a first-line treatment. The results summary can be seen in Table 3.

**Figure 2:**
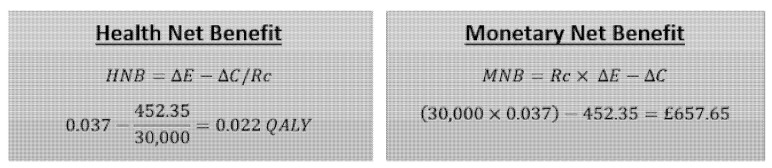
Health and monetary net benefit of treatments.

The health net benefit and monetary net benefit is outlined in Figure 2.

### Sensitivity Analysis

The studies we have evaluated have looked at OAB in an older population. However, it is important to recognize that OAB can affect younger patients in some cases. Unfortunately, there has been little research conducted to evaluate the effects in younger patients as an exclusion criterion of patients under 18 was implemented in our original study. From the data available, the most significant difference observed between the over 18 and under 18 populations were the probabilities of oxybutynin therapy being more efficacious in younger people [42]. Therefore, with this information, we conducted a sensitivity analysis to see the impact of such a significant change. This demonstrated that Botox was not cost-effective. Hence, if Botox were to be implemented as a first-line treatment, more research needs to be conducted to evaluate if there should be an age threshold implemented, and that this may not apply to younger patients who respond better to oxybutynin therapy. Sensitivities of the treatments are included in Table 4.

The percentage of people who experienced dry mouth varied a lot in the literature, with some values as high as 80% [43]. Oxybutynin had a high dropout rate due to adverse effects, and an increase in side effects was expected to have a drastic impact on the ICER. Therefore, for our second sensitivity analysis, we changed the probability of dry mouth from 37% to 80%. Sensitivities at 80& are shown in Table 5.

The utility decreased significantly, as when more people discontinue treatment, they returned to their baseline probability of incontinence episodes, which have a lower average utility. Furthermore, when there are adverse effects, this pushes more people to switch treatments to Botox. Therefore, despite the complication of dry mouth not having a cost tied to it, the change in proportion still has a huge impact on overall cost of treatment from £611.19 to £699.12, a percentage increase of 14.4%.

The ICER increased to £19,761.85 when using the lowest probability of oxybutynin causing dry mouth (7%) as shown in Table 6 [44]. Despite the knock-on effects of having greater treatment adherence and higher average utilities, the ICER still remained below the NICE threshold. This suggests that Botox is a cost-effective treatment for adults with OAB.

## 4. Discussion

**Figure 3:**
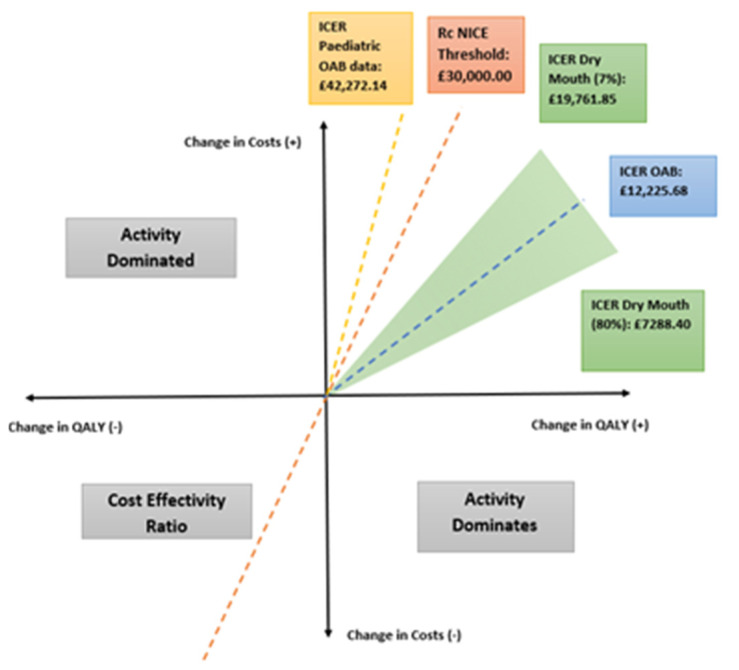
Cost-effectiveness.

Our results show an ICER below the threshold value of £30,000 and a positive MNB and HNB. This is in line with current literature reviewing the use of Botox in OAB conducted from the US perspective. Oxybutynin is the first-line antimuscarinic that is NICE-recommended [7]. It is currently one of the better tolerated and more costeffective antimuscarinics available. Therefore, it can be safely concluded that Botox is more cost-effective than all other drugs in the antimuscarinic class for OAB. When altering the probability of dry mouth in our decision tree, the ICER remained smaller than the threshold at the upper and lower threshold, suggesting that Botox was still more cost-effective than oxybutynin. Figure 3 shows that the ICERS lie in the top right quadrant as this is where there is a higher cost but more efficacious treatment.

**Figure 4:**
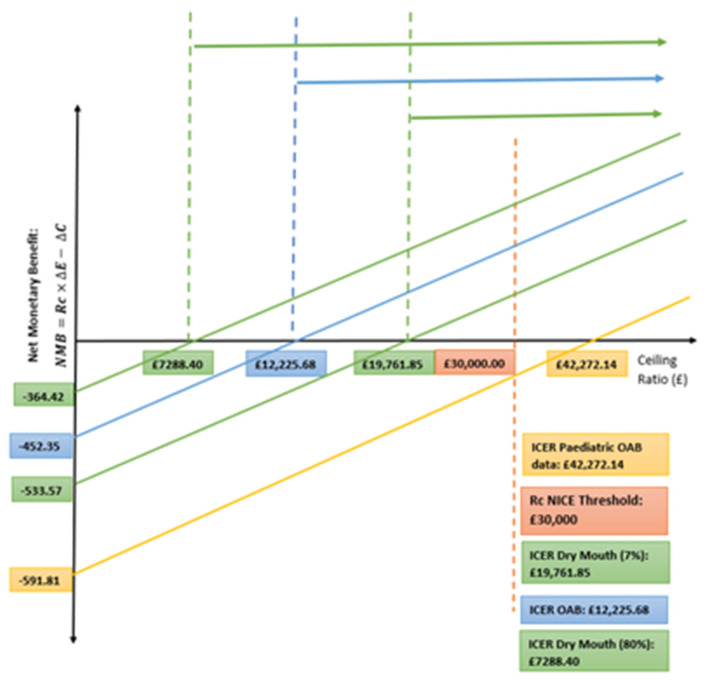
Net Monetary benefit.

The pediatric sensitivity suggests that Botox would not be cost-effective for younger patients with OAB, as this ICER line is beyond the NICE threshold of £30,000 and has a negative NMB, unlike the rest which show positive NMB values, demonstrated in Figure 4. The arrows in the graph represent the area where the intervention becomes cost-effective, i.e., where the intervention crosses the x axis and the MNB becomes positive. As the pediatric sensitivity analysis crosses the x axis, to the right of the £30,000 NICE threshold, it is not cost-effective.

In terms of feasibility, Botox injections are outpatient procedures, which requires patients to travel farther to their appointments. As OAB shows a disease in the older population, many struggle to travel without assistance, resulting in increased difficulties associated with this. Therefore, to address this, an analysis from the patient’s perspective should be conducted to appreciate the full picture of a patient with this condition. Both original studies used were double-blind randomized control trials, increasing the demographic generalizability aspects of the data. The majority of costs were taken directly from NHS tariffs or the BNF from making them generalizable across the entirety of the UK. The utility data were sourced from an amalgamation of two papers, increasing its generalizability to the UK.

### 4.1. Limitations

If oxybutynin is not tolerated, alternative antimuscarinics or mirabegron would be offered before Botox is used. This brings the dilemma of combination treatment being used for OAB, which is out of the scope of this analysis and therefore was excluded when making our tree. A more comprehensive analysis including combinations should be conducted to add further clarification to our findings. The discontinuation rate of Botox is 1.4% and was not included when making the decision tree in the study. This is because most discontinuations occur after the second treatment of Botox [22]. As it is recommended that the Botox treatments occur every 6 months, this would fall outside the time span of the tree, (1 year) and therefore was not included [31].

The probabilities were collected from five different studies [22,23,31,32,33]. In the Botox study [22], the base case values of UIE’s were a lot worse, i.e., people were more likely to be having more UIE’s, and therefore, compared to antimuscarinics, the final outcome was more likely to be worse, resulting in an overestimation of the ICER. Furthermore, 65.4% of people who use Botox are dry by the time they have re-injected [23]. The rest of the probabilities were taken to be the same proportions as the Botox, due to lack of data. This can be justified as those whose reinjection did not completely alleviate their symptoms are likely to remain at the same level as they were at previously.

We only included one re-injection in the CUA as, in the time frame of one year, there will only be one re-injection. However, over a longer period there will be more, which have not been accounted for. Despite the ICER being positive for the time frame of one year, re-injections are expensive, and over a long period of time may make Botox cost adverse. Ideally, more research should be conducted on OAB long term, so a more accurate evaluation over a long period of time can be conducted.

Despite many attempts to source treatment cost information from the same source, this was impossible to achieve. As a result, additional resources including published scientific articles were used to construct the decision tree. Although this method afforded a complete view of the comparison, we accept that use of varying resources may introduce bias to the financial cost for all interventions.

### 4.2. Contribution to the Literature

Despite in-depth clinical evaluations of Botox in OAB, most of the literature does not analyze the costs associated with the procedure. Although the US study [23] highlighted the superiority of using Botox as a first-line treatment, there has been no attempt to analyze this from a UK perspective. This study is congruent with the available literature and suggests that Botox should be implemented as a first-line treatment for OAB.

## 5. Conclusions

This CUA demonstrates the cost-effectiveness of Botox, making it superior to antimuscarinics with an ICER of £12,225.68 spent per QALY gained. When changing the probability of dry mouth, the ICER remained cost-effective which suggests that Botox remains a cost-effective option for OAB. However, the analysis of a pediatric demographic (a minority of OAB patients) showed that Botox was not cost-effective due to better responsiveness of drug therapy at younger age groups. More research needs to be conducted to evaluate at what age Botox should be implemented. Furthermore, a greater range of sensitivity analyses should be conducted to fully confirm the cost-effectiveness of Botox.

## Figures and Tables

**Table 1:** Cost of Treatments.

Treatment	Cost
Botox	£597.56
Botox Reinjection	£407.40
Oxybutynin	£273.39
Constipation	£129.47
Dry Mouth	£0.00
UTI	£94.38
Urinary Retention	£75.39
Level 1 Pad Use	£76.28
Level 2 Pad Use	£152.55
Level 3 Pad Use	£228.83
Level 4 Pad Use	£533.92

**Table 2:** Cost and utility values of complications.

Level	Number of Urinary Incontinence Episodes	Utilities
**1/Dry**	0	0.915
**2**	1–2	0.853
**3**	3–5	0.796
**4**	5+	0.767

Utility = EQ5D score of each incontinence level (with the disutility of any side effects accounted for) x by 1 (length of trial in years).

**Table 3:** Results summary.

	Botox	Oxybutynin
**Total Cost (£)**	£1063.54	£611.19
**Total Utility (QALY)**	0.859	0.822
**ICER**	$\frac{COSTbotox-COSToxy}{EFFECTbotox-EFFECToxy}=\frac{\Delta C}{\Delta E}=\frac{1063.54-611.19}{0.859-0.822}=£12,225.68$	

**Table 4:** Sensitivities of Botox and oxybutynin.

SENSITIVITY:	Botox	Oxybutynin
**Total Cost (£)**	£1063.54	£471.73
**Total Utility (QALY)**	0.859	0.845
**ICER**	$\frac{COSTbotox-COSToxy}{EFFECTbotox-EFFECToxy}=\frac{\Delta C}{\Delta E}=\frac{1063.54-471.73}{0.859-0.845}=£42,272.14$	

**Table 5:** Sensitivities of Botox and oxybutynin at 80%.

SENSITIVITY at 80%:	Botox	Oxybutynin
**Total Cost (£)**	£1063.54	£699.12
**Total Utility (QALY)**	0.859	0.809
**ICER**	$\frac{COSTbotox-COSToxy}{EFFECTbotox-EFFECToxy}=\frac{\Delta C}{\Delta E}=\frac{1063.54-699.12}{0.859-0.809}=£7288.40$	

**Table 6:** Sensitivities of Botox and oxybutynin at 7%.

SENSITIVITY at 7%:	Botox	Oxybutynin
**Total Cost (£)**	£1063.54	£529.97
**Total Utility (QALY)**	0.859	0.832
**ICER**	$\frac{COSTbotox-COSToxy}{EFFECTbotox-EFFECToxy}=\frac{\Delta C}{\Delta E}=\frac{1063.54-529.97}{0.859-0.832}=£19,761.85$	

## Data Availability

All data utilized is referenced and available within the manuscript or the Appendix A, Appendix B, Appendix C, Appendix D, Appendix E and Appendix F.

## Supplementary Materials

The following are available online at https://www.mdpi.com/article/10.3390/ijerph18168743/s1, Table S1: Decision Tree.

## Appendix A

**Table A1:** Costs For Botox.

Component	Cost	Discount Rate	Cost with Discount	Reference
**Botox Vial**	£138.20	N/A	£138.20	(NICE BNF, 2021a [26])
**Botox** **administration**	£219.00	1.035^6^	(£219.00 × 1.035^6^) = £269.20	OnabotulinumtoxinA in the treatment of overactive bladder (Freemantle et al., 2016 [22])
**GP Visit**	£62.70	1.035^5^	(62.70 × 1.035^5^) = £74.47	Unit Costs of Health and Social Care 2016 (PSSRU, 2016 [30])
**Urology outpatient visit**	108	1.035^2^	(108 × 1.035^2^) = £115.69	2018/2019 National cost collection data of NHS (NHS England, 2018 [45])
**TOTAL**	£138.20 + 269.20 + 74.47 + £115.69 = £597.56			£597.56

**Table A2:** Cost For Botox Re-injection.

Component	Cost	Discount Rate	Cost with Discount	Reference
**Botox Vial**	£138.20	N/A	£138.20	(NICE BNF, 2021a [26])
**Botox** **administration**	£219.00	1.035^6^	(£219.00 × 1.035^6^) = £269.20	OnabotulinumtoxinA in the treatment of overactive bladder (Freemantle et al. N, 2016 [22])
**TOTAL**	£138.20 + 269.20 = £407.40			£407.40

**Table A3:** Cost For oxybutynin.

Component	Cost	Discount Rate	Cost with Discount	Reference
**Oxybutynin** **treatment**	£3.00 per month	N/A	(3.00 × 12) = £36.00	(NICE BNF, 2021b [27])
**GP Visit**	£62.70	1.035^5^	(62.70 × 1.035^5^) = £74.47	Unit Costs of Health and Social Care 2016 (PSSRU, 2016 [30])
**GP maintenance visits**	£42.60	1.035^3^	(£42.60 × 1.035^3^) = £47.23	Cost-effectiveness of solifenacin compared with oral antimuscarinic agents (Hakimi et al., 2018 [32])
**Urology outpatient visit**	108	1.035^2^	(108 × 1.035^2^) = £115.69	2018/2019 National cost collection data of NHS (NHS England, 2018 [45])
**TOTAL**	£36.00 + £115.69 + £74.47 + £47.23 = £273.39			£273.39

**Table A4:** Cost For incontinence pads.

Component	Cost	Discount Rate	Cost with Discount	Reference
**Incontinence Pads: Dry**	£0.17	1.035^6^	365 × 0.17 × 1.035^6^ = £76.28	OnabotulinumtoxinA in the treatment of overactive bladder (Freemantle et al., 2016 [22])
**Incontinence Pads: 0–2**	£0.17 × 2	1.035^6^	365 × 0.17 × 2 × 1.035^6^ = £152.55	
**Incontinence Pads: 3–5**	£0.17 × 3	1.035^6^	365 × 0.17 × 3 × 1.035^6^ = £228.83	
**Incontinence Pads: 5+**	£0.17 × 7	1.035^6^	365 × 0.17 × 7 × 1.035^6^ = £533.92	

## Appendix B

**Table A5:** Cost Of Complications.

Component	Cost	Discount Rate	Cost with Discount	Reference
**Urinalysis**	£19	N/A	£19	(NIHR, 2020 [45])
**UTI treatment:** **physician time**	£62.70	1.035^5^	(62.70 × 1.035^5^) = £74.47_(10)_	Unit Costs of Health and Social Care 2016 (PSSRU, 2016 [30])
**UTI treatment:** **Trimethoprim 200 mg**	£1.27 14 pills	N/A	£1.27/14 × 10 = £0.91 200 mg BD 5 days	(NICE BNF, 2021d [28])
**TOTAL UTI PRICE:**	£74.47 + £0.91 + £19 = £94.38			£94.38
**Constipation: Senna tablets**	£2.20/60 tablets 25 packs a year->4 tablets a day	N/A	2.20 × 25 = £55.00	(NICE BNF, 2021c [29])
**Constipation:** **Physician time**	£62.70	1.035^5^	(62.70 × 1.035^5^) = £74.47_(10)_	Unit Costs of Health and Social Care 2016 (NHS England, 2016 [45])
**Constipation:** **Total Cost**	£55.00 + £74.47 = £129.47			£129.47
**Dry Mouth: Total Cost**	£0	N/A	N/A	There is no prescription treatment for dry mouth.
**Urinary retention** **Catheter cost^22^**	£0.75	1.035^6^	£0.75 × 1.035^6^ = £0.92	OnabotulinumtoxinA in the treatment of overactive bladder (Freemantle et al., 2016 [22])
**Urinary retention: GP appointment**	£62.70	1.035^5^	(62.70 × 1.035^5^) = £74.47_(10)_	Unit Costs of Health and Social Care 2016 (PSSRU, 2016 [30])
**Urinary retention: Total Cost**	£74.47 + £0.92 = £75.39			£75.39

## Appendix C

**Table A6:** Probabilities used in the decision tree.

Component	Probability	Reference	Comments/Limitations
**Reinjection of** **Botox**	Yes: 0.667 No:0.333	OnabotulinumtoxinA in the treatment of overactive bladder (Freemantle et al., 2016 [22])	
**Treatment Switch to Botox: with no** **adverse effects**	No: 0.982 Yes: 0.018	Patterns of use of antimuscarinic drugs to treat overactive bladder (Margulis et al., 2018 [31])	
**Treatment Switch to Botox: with adverse effects**	No:0.766 Yes: 0.234	Patterns of use of antimuscarinic drugs to treat overactive bladder (Margulis et al., 2018 [31])	
**Treatment Discontinuation with adverse effects**	Yes:0.9 No:0.1	Cost-effectiveness of solifenacin compared with oral antimuscarinic agents (Hakimi et al., 2018 [32])	
**Treatment Discontinuation without adverse effects**	Yes:0.064 No:0.936	Cost-effectiveness of solifenacin compared with oral antimuscarinic agents (Hakimi et al., 2018 [32])	
**Urinary Tract Infection**	Yes: 0.204 No: 0.796	OnabotulinumtoxinA in the treatment of overactive bladder (Freemantle, 2016 [22])	
**Urinary Retention**	Yes: 0.069 No: 0.931	OnabotulinumtoxinA in the treatment of overactive bladder (Freemantle, 2016 [22])	
**Dry Mouth**	Yes: 0.37 No: 0.63	Cost-effectiveness of solifenacin compared with oral antimuscarinic agents (Hakimi et al., 2018 [32])	Extreme values were found for this point e.g., 80%, and 7%_._ Which is why we conducted a sensitivity analysis.
**Constipation**	Yes: 0.037 No:0.963	Cost-effectiveness of solifenacin compared with oral antimuscarinic agents (Hakimi et al., 2018 [32])	
**Probability of Level 1–4 UIE for discontinued oxybutynin**	(1) 0.0 (2) 0.178 (3) 0.351 (4) 0.356	OnabotulinumtoxinA in the treatment of overactive bladder (Freemantle el 2016 [22])	The probabilities of people having UIE episodes after failed anticholinergic use, without further treatment.
**Probability of Level 1–4 for oxybutynin**	(1) 0.23 (2) 0.367 (3) 0.191 (4) 0.212	A Comparative Review of Oxybutynin Chloride Formulations (Kennelly, 2010 [33])	The probability of 23% was found from this article. The remaining probabilities of levels 2–4 were calculated on the assumption that they followed the same proportions as the Botox data.
**Probability of Level 1–4 for Botox**	(1) 0.289 (2) 0.339 (3) 0.176 (4) 0.196	OnabotulinumtoxinA in the treatment of overactive bladder (Freemantle et al., 2016 [22])	
**Probability of Level 1–4 for reinjected Botox**	(1) 0.654 (2) 0.166 (3) 0.085 (4) 0.095	Interstim Sacral Neuromodulation and Botox Botulinum-A Toxin Intradetrusor Injections for Refractory Urge Urinary Incontinence (Shepherd, 2011 [23])	The probability of 65% was found from this article. The remaining probabilities of levels 2–4 were calculated on the assumption that they followed the same proportions as the Botox probabilities.

## Appendix D

**Table A7:** Output Utilities.

Component	Utility	Reference	Comments
**Level 1: Dry**	0.915	OnabotulinumtoxinA in the treatment of overactive bladder (Freemantle et al., 2016 [22])	These utilities are the standardized utilities at the EQ5D for that number of UIE’s for 1 year. The antimuscarinic trials output was measured in 5 incontinence levels which could be converted to this more intuitive level system. Therefore, we did this and made the assumption that the utilities would be the same.
**Level 2: 1–2**	0.853		
**Level 3: 3–5**	0.796		
**Level 4: 5+**	0.767		
**Disutility per side effect**	−0.036	Cost-effectiveness of solifenacin compared with oral antimuscarinic agents (Hakimi et al., 2018 [32])	This was based on dry mouth and constipation. We assumed the same disutility could be applied to urinary retention and urinary tract infection.

## Appendix E

**Table A8:** Sensitivity Analysis Utility Change For Paediatric OAB.

Level	Number of Urinary Incontinence Episodes	Probability of Each Level for Oxybutynin-Adult	Probability of Each Level for Oxybutynin-Children.
**1/Dry**	0	0.23	0.61
**2**	1–2	0.367	0.185
**3**	3–5	0.191	0.097
**4**	5+	0.212	0.108

## Appendix F

**Table A9:** Inclusion and exclusion criteria for literature search.

Inclusion Criteria	Exclusion Criteria
Written in English	Written in language other than English
Articles specifically referring to overactive bladder syndrome	Articles referring to non-OAB conditions e.g., UTIs
Articles focusing on antimuscarinic/anticholinergic/Botox treatments	Articles focusing on treatment plans that do not include antimuscarinics/anticholinergics/Botox
5+ Systematic reviews, RCTs, meta-analysis, CEA, CUA, CBA	Qualitative studies
